# A systematic review comparing the costs of chiropractic care to other interventions for spine pain in the United States

**DOI:** 10.1186/s12913-015-1140-5

**Published:** 2015-10-19

**Authors:** Simon Dagenais, O’Dane Brady, Scott Haldeman, Pran Manga

**Affiliations:** Spine Research LLC, 540 Main Street #7, Winchester, MA 01890 USA; World Spine Care, Santa Ana, CA USA; Department of Neurology, College of Medicine, University of California, Irvine, USA; Department of Epidemiology, School of Public Health, University of California, Los Angeles, USA; Telfer School of Management, University of Ottawa, Ottawa, Ontario Canada

**Keywords:** Chiropractic, Spine pain, Cost comparison, Economic evaluation, United States

## Abstract

**Background:**

Although chiropractors in the United States (US) have long suggested that their approach to managing spine pain is less costly than other health care providers (HCPs), it is unclear if available evidence supports this premise.

**Methods:**

A systematic review was conducted using a comprehensive search strategy to uncover studies that compared health care costs for patients with any type of spine pain who received chiropractic care or care from other HCPs. Only studies conducted in the US and published in English between 1993 and 2015 were included. Health care costs were summarized for studies examining: 1. private health plans, 2. workers’ compensation (WC) plans, and 3. clinical outcomes. The quality of studies in the latter group was evaluated using a Consensus on Health Economic Criteria (CHEC) list.

**Results:**

The search uncovered 1276 citations and 25 eligible studies, including 12 from private health plans, 6 from WC plans, and 7 that examined clinical outcomes. Chiropractic care was most commonly compared to care from a medical physician, with few details about the care received. Heterogeneity was noted among studies in patient selection, definition of spine pain, scope of costs compared, study duration, and methods to estimate costs. Overall, cost comparison studies from private health plans and WC plans reported that health care costs were lower with chiropractic care. In studies that also examined clinical outcomes, there were few differences in efficacy between groups, and health care costs were higher for those receiving chiropractic care. The effects of adjusting for differences in sociodemographic, clinical, or other factors between study groups were unclear.

**Conclusions:**

Although cost comparison studies suggest that health care costs were generally lower among patients whose spine pain was managed with chiropractic care, the studies reviewed had many methodological limitations. Better research is needed to determine if these differences in health care costs were attributable to the type of HCP managing their care.

**Electronic supplementary material:**

The online version of this article (doi:10.1186/s12913-015-1140-5) contains supplementary material, which is available to authorized users.

## Background

Spine pain is one of the most common and costly causes of health care utilization in the United States (US), with 61 % of patients seeking care from a medical physician (i.e. medical doctor (MD) or doctor of osteopathy (DO)), 28 % from a chiropractor, and 11 % from both a medical physician and a physical therapist (PT) [1–4]. Chiropractors in the US treat spine pain almost exclusively, with the most common indication for care being low back pain (LBP) (68 %), followed by neck pain (12 %), and mid-back pain (6 %) [5]. By contrast, only 3 % of office visits to medical physicians are related to spine pain [6].

**Fig. 1:**
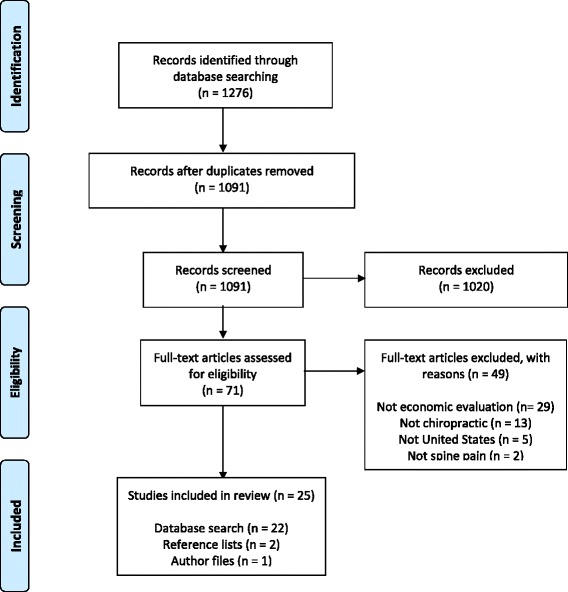
Study flow diagram

Studies have reported that chiropractors have more confidence in their ability to manage spine pain than medical physicians, and that patients with spine pain report higher levels of satisfaction with chiropractic care than care from a medical physician [7–9]. Proponents of chiropractic maintain that it offers a more cost-effective approach to managing spine pain for a variety of reasons, including lower fees for office visits, use of x-rays rather than more advanced diagnostic imaging, lower referral rates to spine specialists or surgeons, and scope of practice limitations related to medications, injections, and surgery [10].

Previous reviews have examined the cost effectiveness of chiropractic care for occupational LBP, spinal manipulation therapy (SMT) for spine pain, treatments endorsed by clinical practice guidelines (CPGs) for LBP, conservative care for neck pain, and complementary and alternative medical (CAM) therapies for spine pain [11–17]. However, such reviews included a multitude of therapies and countries, and were therefore not focused on chiropractic care for spine pain in the US.

The primary objective of this study was to systematically review cost comparison studies examining chiropractic care for spine pain in the US.

## Methods

### Literature search

This review was conducted in accordance with the PRISMA guidelines for reporting systematic reviews [18]. A broad search combining indexed search terms and free text search terms related to chiropractic care (developed by the authors), spine pain (adapted from the Cochrane Back Review Group search strategy), and cost comparison studies (developed by the authors) was undertaken in August 2013 and an updated search was performed in August 2015 using the OvidSP interface for the Medline, Embase, NHS Economic Evaluation Database (EED), and Health Technology Assessment (HTA) databases. Additional searches were conducted in the CEA registry (https://research.tufts-nemc.org/cear4/), Index to Chiropractic Literature (ICL) (http://www.chiroindex.org/), and EconLit (American Economic Association) (https://www.aeaweb.org//econlit/efm/index.php) databases. References from previous related reviews and author files were also searched. The search strategy used in Medline is included in Additional file 1; others are available upon request [19, 20].

### Inclusion criteria

Studies that met all of the following criteria were deemed eligible for this review:

1. At least one study group received chiropractic care (i.e. care provided by a chiropractor, regardless of the interventions used, since these often vary or are not specified in study protocols);
2. At least one study group did not receive chiropractic care, or study design otherwise allowed for comparison of chiropractic care to another approach (e.g. study comparing medical care to medical care and chiropractic care);
3. Primary condition treated was spine pain (i.e. neck pain, mid-back pain, or LBP with or without red flags suggestive of serious pathology);
4. Health care costs were reported in both study groups;
5. Study was performed in the United States;
6. Study was published as a full-text journal manuscript in English.

### Exclusion criteria

Studies that met any of the following criteria were deemed ineligible for this review:

1. Chiropractic therapy not performed by a chiropractor (e.g. SMT by a MD);
2. Review article without original data;
3. Abstracts, conference proceedings, presentations;
4. Published prior to 1993.

### Study screening

The combined search results were screened independently by two reviewers (SD and OB) based on the search records to determine relevance. Disagreements were discussed until consensus was reached. Full-text manuscripts were obtained for studies deemed relevant or of unclear relevance.

### Data extraction and analysis

The following data were extracted by one reviewer (SD) and verified by another reviewer (OB):

1. Study design (e.g. location, data type, source, and dates, population size);
2. Study indication (e.g. duration, inclusion criteria, exclusion criteria, diagnoses);
3. Study groups (e.g. number and size of groups, therapy or provider involved);
4. Health care and other costs (e.g. scope of costs, episodes, cost containment);
5. Health care utilization (e.g. imaging, medications, hospitalization, surgery);
6. Clinical outcomes data (e.g. pain severity, physical function, quality of life).

Data comparing health care costs and other costs were summarized to compare findings for study groups that received care from a doctor of chiropractic (DC) to those who received care from any other type of HCP. When data were reported for multiple subgroups of patients (e.g. different categories of spine pain), they were combined to report data for the entire study group (e.g. all patients with spine pain). Data from study groups that received both chiropractic care and care from other HCPs were assigned to the comparator group (i.e. if a study compared chiropractic care to care from both a medical physician and a chiropractor, the latter group was assigned as a comparator).

### Study quality assessment

Although instruments such as the Consensus on Health Economic Criteria (CHEC) list are available to assess the methodological quality of cost effectiveness analyses and cost utility analyses, they are not readily applicable to cost comparison studies in which clinical outcomes are not measured [21]. A modified version of the CHEC list omitting item 14 (deemed not applicable since none of the studies projected health care costs into the future) was used to assess the methodological quality of cost comparison studies also examining clinical outcomes.

## Results

### Search

The search strategy returned 1276 citations, including 530 (42 %) from Embase, 344 (27 %) from ICL, 153 (12 %) from Medline, 88 (7 %) from the CEA registry, 67 (5 %) from NHS EED, 61 (5 %) from EconLit, and 33 (3 %) from NHS HTA. Upon combining these results, a total of 185 duplicate citations (15 %) were uncovered and removed, yielding 1091 unique citations. Screening determined that 1,020 (94 %) of these 1091 citations were not relevant; full-text articles were obtained for 71 (7 %) citations. Upon reviewing 71 full-text articles, 29 (41 %) were excluded because they were not cost comparison studies, 13 (18 %) were not related to chiropractic care, 5 (7 %) were not conducted in the US, and 2 (3 %) were not related to spine pain (note: only the primary reason for exclusion was noted; studies could be excluded for multiple reasons). In addition to the 22 (88 %) studies identified by screening search records from electronic databases, 2 (8 %) studies were identified from reference lists of previous reviews, and 1 (4 %) study was identified from author files, yielding a total of 25 eligible studies for this review (Fig. 1).

### Study characteristics

Twelve (48 %) studies examined data from private health plans in the US [22–33]. Six (24 %) studies examined data from worker’s compensation (WC) plans in the US [34–39]. Seven (28 %) studies compared both health care costs and clinical outcomes [40–46]. Findings from these three groups of studies are presented below.

### Cost comparison studies in private health plans

#### Study design

Overall study design for the twelve studies in this group is summarized in Table 1. All studies were retrospective. Eight studies examined data from fee for service health plans, two examined health maintenance organizations (HMOs) (one study did not specify health plan type), and one study examined data from a self-funded employer. Eight studies reported the total number of members in the health plans examined, which ranged from 7706 to 2 million, while four did not report this information. Nine studies examined health care costs using 24 months of claims data, one used 12 months of data, and one used 48 months of data. Eight studies considered only LBP (as defined by 4–12 ICD-9 codes), while three included other regions of spine pain (as defined by 58–82 ICD-9 codes).

**Table 1:** Study design for cost comparisons in private health plans

First author	Year	Location/Plan type	Data type/Source	Data start -Data end	Eligibility	Indication/Duration	# ICD-9 codes	Inclusion	Exclusion	# Insured/# Eligible/# Included
Allen [33]	2014	National/Self-insured employer	Claims/Navistar integrated database^e^	1/1/2001 - 12/31/2009	Back pain diagnosis	Low back pain/Any	77	1. Back pain episodes identified by one or more of 77 ICD-9 codes 2. 6 months of recorded continuous coverage	Cancer diagnosis, retired, died during study period	NR/21,080/14,787^d^
Grieves [29]	2009	Wisconsin/HMO	Claims/Arise Health Plan	1/1/2004 - 12/31/2005	1. Continuously enrolled, and 2. Claim for visit to MD or DC for 12 ICD-9 codes	Low back pain/Any	12	1. Continuously enrolled; 2. Any claim for visit to MD or DC	Saw both MD and DC	30,000/NR/897
Liliedahl [32]	2010	Tennessee/NR	Claims/BlueCross BlueShield	10/1/2004 - 9/30/2006	1. Claim for 82 ICD-9 codes related to spine pain, and 2. CPT code for visit to MD, DO, ED, or DC	Spine pain/Any	82	CPT code for visit to MD, DO, ED, or DC	Episodes not related to MD, DO, ED, or DC, or with incomplete claims records	669,320/85,402/102,438^d^
Mosley [30]	1996	Louisiana/HMO	Claims/Community Health Network of Louisiana	10/1/1994 - 10/1/1995	Claim for ICD-9 codes 720.0 to 724.9 (potentially 68 different ICD-9 codes) related to back or neck pain	Back or neck pain/Any	68	Claims related to ICD-9 codes	NR	NR/1,959/1,959
Shekelle [31]	1995	National/Fee for service	Claims/RAND Health Insurance Experiment	NR (mean 3.6 months)^b^	Claim related to pain, swelling, or injury of back region	Pain, swelling, or injury of back/Any	NA	Any back-related claim as 1/2/3 symptom	1.Elderly; 2.Care paid by workers’ compensation	7,706/686/1,020^d^
Smith [27]	1997	National/Fee for service	Claims/MEDSTAT	7/1/1988 - 6/30/1990	10 % sample of claims for 493 ICD-9 codes related to MSK conditions or claims for chiropractic care	Low back conditions/Any	9	10 % sample with claim related to ICD-9 codes	Unknown first-contact provider type	2,000,000^a^/434,763/890
Stano [28]	1996	National/Fee for service	Claims/MEDSTAT	7/1/1988 - 6/30/1990	10 % sample of claims for 493 ICD-9 codes related to MSK conditions or claims for chiropractic care	Low back conditions/Any	9	10 % sample with claim related to ICD-9 codes	1. Unknown first-contact provider type; 2. Negative value claims	2,000,000^a^/434,763/6,183
Stano [23]	1995	National/Fee for service	Claims/MEDSTAT	7/1/1988 - 6/30/1990	10 % sample of claims for 493 ICD-9 codes related to MSK conditions	Low back conditions/Any	9	10 % sample with claim related to ICD-9 codes	Negativepayments	NR/434,763/6,799
Stano [24]	1994	National/Fee for service	Claims/MEDSTAT	7/1/1988 - 6/30/1990	Claim for 493 ICD-9 codes related to MSK conditions	Low back conditions/Any	15	Claim related to ICD-9 codes	1. Coinsurance >15 %; 2. Deductibles > $200; 3. No restrictions on chiropractic or medical care	NR/395,641/42,331
Stano [22]	1993	National/Fee for service	Claims/MEDSTAT	7/1/1988 - 6/30/1990	Claim for 493 ICD-9 codes related to MSK conditions	Low back conditions/Any	15	Claim related to ICD-9 codes	1. Did not meet deductibles; 2. Did not file claims; 3. Out-of-plan use; 4. Age >65	2,000,000^a^/395,641/99,675
Stano [25]	1993	National/Fee for service	Claims/MEDSTAT	7/1/1988 - 6/30/1990	Claim for 493 ICD-9 codes related to MSK conditions	Low back conditions/Any	9	10 % sample with claim related to ICD-9 codes	NR	2,000,000^a^/396,000^a^/7,880
Stano [26]	1993	National/Fee for service	Claims/MEDSTAT	7/1/1988 - 6/30/1990	1. Claim for 493 ICD-9 codes related to MSK conditions, or 2. Claim for chiropractic care	Low back conditions/Any	4	Claim related to ICD-9 codes	1. Did not meet deductibles; 2. Did not file claims; 3. Out-of-plan use; 4. Elderly	2,000,000^a^/396,000^a^/10,945

^a^number reported as estimated

^b^as reported in separate study, 70 % participated for 36 months and 30 % participated for 60 months [64]

^c^insured n and eligible n reported only for studies based on claims. Insured n refers to size of insured population. Eligible n refers to size of insured population meeting stated eligibility criteria

^d^refers to the number of episodes of spine pain (i.e. members could have multiple episodes)

^e^as reported in separate study [65]

*CPT* current procedural terminology, *DC* doctor of chiropractic, *DO* doctor of osteopathy, *ED* emergency department, *HMO* health maintenance organization, *ICD* International Classification of Diseases, *LBP* low back pain, *MD* medical doctor, *MSK* musculoskeletal, *NA* not applicable, *NR* not reported, *RAND* Research and Development Corporation

Seven studies compared health care costs for episodes of care that began with a claim for one of the ICD-9 codes of interest, six of these seven studies ended claims with a period of 35–90 days without care; five studies did not define episodes of care. Eight studies assigned all health care costs for an episode of care to the first HCP to submit a claim, while one assigned costs to the HCP who delivered the majority of care; three did not specify how they assigned costs. Six studies compared health care costs for all claims during an episode of care, while four considered only claims related to spine pain; two did not specify this. One study had five comparator groups (care from a medical physician general practitioner, internist, doctor of osteopathy, orthopedist, or other types of HCPs), one study had four comparator groups (care from a medical physician, information and advice, physical therapy, or multiple types of HCPs), one study had two comparator groups (care from a primary care medical physician or specialist), and eight studies had only one comparator (care from a medical physician). Few details were provided about the care received from different HCPs (e.g. therapies, protocols, frequency of care).

#### Cost comparison

Cost comparison findings for the twelve studies in this group are summarized in Table 2. Seven studies included three or more categories of health care costs (e.g. outpatient, inpatient, medications, imaging) in their comparisons, while three compared health care costs without defining the specific costs included, and two compared only outpatient health care costs (no definition provided). Ten studies compared costs paid by the health plan (i.e. costs allowed minus patient copay, coinsurance, deductible, etc.), while one compared costs allowed, and one compared costs billed by HCPs. Seven studies analyzed costs for each member with spine pain, while five analyzed costs by episode of care for spine pain (i.e. members could have multiple episodes of care). The number of members/episodes included in groups receiving chiropractic care ranged from 97 to 36,280 (mean 5149, standard deviation (SD) 10,222, median 1624), while in comparator groups it ranged from 101 to 66,158 (mean 11,456, SD 18,764, median 4910). The costs of health care for spine pain by member/episode who received chiropractic care ranged from $264 to $6,171 (mean $2,022, SD $2,332, median $712), while in comparator groups it ranged from $166 to $9,958 (mean $3,375, SD $3,481, median $1,992). In eleven (92 %) studies, health care costs were lower for patients whose spine pain was managed with chiropractic care. The difference in health care costs for members who received chiropractic care ranged from −70 % to 59 % (mean −36 %, SD 33 %, median −38 %).

**Table 2:** Comparison of health care costs in private health plans

				Chiropractic care		Comparator group(s)	Comparator			Lowest	Difference^c^
First author	Year	Health care costs included	Cost type	*N*	Costs		*n*		Costs		
Allen [33]	2014	Outpatient (DC, MD, PT), medications, surgery, imaging, injections, other	Paid	1672^a^	$6,171	1. MD care, 2. advice, 3. PT, 4. multiple providers	13,115^a^		$9,958	Chiropractic	−38 %
Grieves [29]	2009	Imaging, hospital, physical therapy, outpatient office	Allowed	411	$851	1. MD primary care, 2. MD specialist care	486		$2,871	Chiropractic	−70 %
Liliedahl [32]	2010	All health care services	Paid	36,280	$452	MD care	66,158		$740	Chiropractic	−39 %
Mosley [30]	1996	Imaging, medications, other	Paid	121	$539	MD care	1,838		$774	Chiropractic	−30 %
Shekelle [31]	1995	Hospital care, physician services, medications, outpatient services, injections, supplies	Billed	412^a^	$264	1. MD primary care, 2. orthopedist, 3. internist, 4. DO, 5. other providers	608^a^		$166	Comparator	59 %
Smith [27]	1997	Not specified	Paid	97^b^	$1,038	MD care	101^b^		$3,068	Chiropractic	−66 %
Stano [28]	1996	Not specified	Paid	1,575^a^	$518	MD care	4,608^a^		$1,020	Chiropractic	−49 %
Stano [23]	1995	Outpatient (not specified)	Paid	2,408^a^	$493	MD care	4,391^a^		$1,000	Chiropractic	−51 %
Stano [24]	1994	Outpatient (not specified)	Paid	10,659	$5,474	MD care	27,021		$8,427	Chiropractic	−35 %
Stano [22]	1993	Outpatient (DC, MD, facility, other), inpatient (MD, hospital, other), and medications	Paid	1,326	$2,150	MD care	7,144		$3,127	Chiropractic	−31 %
Stano [25]	1993	Outpatient (DC, MD) and inpatient (not specified)	Paid	2,668^a^	$573	MD care	5,212^a^		$1,112	Chiropractic	−48 %
Stano [26]	1993	Outpatient (DC, MD) and inpatient (not specified)	Paid	4,156	$5,747	MD care	6,789		$8,240	Chiropractic	−30 %

^a^number of episodes (not patients)

^b^minimum 3 episodes with care from same provider

^c^reported as (−(comparator costs - chiropractic care costs)/comparator costs) × 100 %

*DC* doctor of chiropractic, *MD* medical doctor, *PT* physical therapy

### Cost comparison studies in worker’s compensation plans

#### Study design

Overall study design for the six studies in this group is summarized in Table 3; additional information (i.e. members included in analysis) was obtained from a secondary report [47]. Five studies were retrospective and one was prospective. Two studies examined data from private WC plans, one from a self-insured employer WC plan, one from a quasi-state agency for WC, one from a state WC plan, and one from a nonprofit WC plan. Five studies examined only claims data, while one study also included billing data from HCPs and data collected from patients. Only 1/6 (16 %) studies reported the total number of members covered by the WC plan examined (*n* = 96,627). Two studies examined only claims related to LBP, while four examined claims for all regions of spine pain; only one study reported the ICD-9 codes used to define spine pain.

**Table 3:** Study design for cost comparisons in worker’s compensation plans

First author	Year	Location/Payer type	Data type/Source	Data start -Data end	Indication/Duration	Inclusion	Exclusion	# Insured/# Eligible/# Included
Butler[39]	2010	National/Self-insured employers	1. Claims, 2. Billing, 3. Patients/ASU Healthy Back Study	1/1/1999 - 6/30/2002	Occupational back pain/Any	Incident back injuries	NR	NR/1,831/984^a^
Cifuentes[37]	2011	National/Private insurer	Claims/Liberty Mutual	1/1/2006 – 12/31/2006	Lower back, sacrum, coccyx, multiple trunk Any	1. Nonspecific low back pain cases, and 2. Sprain or strain injury	1. Medical only claims; 2. Previous WC claims; 3. Temporary disability < seven days; 4. <four visits to PT/DC; 5. First visit >14 days after injury; 6. Health maintenance period < seven days	NR/11,420/894
Gilkey [35]	2008	Colorado/Quasi state agency	Claims/Pinnacol Assurance	1/1/2000 - 12/31/2002	Nonspecific low back injury/Any	Closed claims for nonspecific low back injuries	1. Multiple providers; 2. Hospitalization; 3. Surgery	NR/10,262/2,456
Jarvis [38]	1997	Utah/Nonprofit insurer	Claims/Worker Compensation Fund of Utah	1/1/1986 - 12/31/1989	Spine injuries/Any	2.5 % sample of NCCI codes for spine injuries in 1986 or 1989	1. Surgical cases; 2. Cases that crossed over from one provider group to another	NR/80,615/1,568
Johnson [34]	1999	California/Private insurer	Claims/Zenith National Insurance Corp.	10/1/1991 - 8/1/1993	Occupational back pain/Any	Closed back claims	1. Surgical cases; 2. Missing data; 3. Did not receive care from PT or DC; 4. Received care from PT and DC; 5. Permanent total disability cases	NR/844/844
Phelan [36]	2004	North Carolina /Various payers	Claims/North Carolina Industrial Commission	1/1/1975 - 12/31/1994	Lumbar or lumbosacral strain/Any	1. Closed injury claims, and 2. Complete data available	NR	96,627/43,650/43,650

^a^data reported in a secondary study report [47]

*ASU* Arizona State University, *DC* doctor of chiropractic, *NCCI* National Council on Compensation Insurance, *NR* not reported, *PT* physical therapist, *WC* workers’ compensation

Three studies examined only closed claims related to spine pain, two examined claims at least 1 year from the date of onset (% closed claims not reported), and one examined claims at least 2 years from the date of onset (97 % were closed). The number of members who met stated eligibility criteria ranged from 1831 to 80,615 (mean 29,556, SD 32,694, median 11,420). The duration of claims data examined ranged from 12 to 239 months (mean 66.5, SD 85.8, median 38.9). The delay between the end of the data period examined and study publication ranged from 4.3 to 10.0 years (mean 6.9, SD 1.9, median 6.7). One study had five comparator groups (care from a medical physician, care from a PT, care from both a medical physician and PT, other care, or no care), one had four comparator groups (care from a medical physician, care from a medical physician and chiropractor, care from a medical physician and PT, or care from other HCPs), one study had three comparator groups (care from a medical physician, medical physician and chiropractor, and no care), and three studies had one comparator group (care from a medical physician). Few details were provided about the care received from different HCPs (e.g. therapies, protocols, frequency of care). One study compared health care costs for patients who had received chiropractic care during both the disability period (i.e. time when they were not working due to spine pain) and after the disability period (i.e. once they had returned to work, defined as the “maintenance period” in that study) [37].

#### Cost comparison

Cost comparison findings for the six studies in this group are summarized in Table 4. All of the studies considered only health care costs related to spine pain, and all compared the amounts paid by WC plans (i.e. not billed by HCPs). Three studies included four or more categories of health care costs (e.g. office visits to DC/MD/PT, medications, imaging) in their comparisons, while three studies included all health care costs without defining the specific costs included. The number of members/claims included in groups receiving chiropractic care ranged from 54 to 1007 (mean 275, SD 362, median 166), while in comparator groups it ranged from 671 to 10,930 (mean 2988, SD 3966, median 1605). The costs of health care for spine pain by member/claim who received chiropractic care ranged from $415 to $1,296 (mean $817, SD $320, median $777), while in comparator groups it ranged from $264 to $7,904 (mean $2,565, SD $3,137, median $867). In five (83 %) studies, health care costs were lower for patients whose spine pain was managed with chiropractic care. The difference in health care costs for members who received chiropractic care ranged from −91 % to 229 % (mean 4 %, SD 116 %, median −18 %).

**Table 4:** Comparison of health care costs in worker’s compensation plans

			Chiropractic care		Comparator group(s)	Comparator		Lowest	Difference^d^
First author	Year	Health care costs included	*n*	Costs		*n*	Costs		
Butler [39]	2010	Office visits, PT, imaging, medications	54^a^	$1,296	1. MD care, 2 MD care + chiropractic care +/− PT care, 3. MD care + PT care, 4. Other	930^a^	$4,925	Chiropractic	−74 %
Cifuentes [37]	2011	Not specified	159^b^	$415	1. PT maintenance care^c^, 2. MD maintenance care, 3. PT and MD maintenance care, 4. Other maintenance care, 5. No maintenance care	735^b^	$566	Chiropractic	−27 %
Gilkey [35]	2008	Not specified	76	$868	MD care	2,380	$264	Comparator	229 %
Jarvis [38]	1997	All health care costs associated with claim	1,007	$596	MD care	2,279	$658	Chiropractic	−9 %
Johnson [34]	1999	Office visits, PT, imaging, other diagnostic testing, medications, hospital visits	173	$1,044	MD care	671	$1,075	Chiropractic	−3 %
Phelan [36]	2004	Office visits to DC/MD/PT, medications, hospital inpatient, hospital outpatient, supplies	181	$685	1. MD care, 2. MD care + chiropractic care, 3. No MD care or chiropractic care	10,930	$7,904	Chiropractic	−91 %

^a^data reported in secondary report [47]

^b^costs were weekly average health care costs during both disability period and maintenance period

^c^maintenance care defined as receiving any type of health care after the initial period of disability has ended

^d^reported as (−(comparator costs - chiropractic care costs)/comparator costs) × 100 %

*DC* doctor of chiropractic, *MD* medical doctor, *PT* physical therapist

### Cost comparison studies also examining clinical outcomes

#### Study design

Overall study design for the seven studies in this group is summarized in Table 5; additional information (i.e. eligibility criteria) was also obtained from secondary reports [48–50]. Four studies were based on observational (OBS) designs (i.e. comparative cohorts), while three were based on randomized controlled trials (RCTs). Of the four OBS studies, three examined patients with LBP who received care at one of 51 chiropractic clinics and 14 medical clinics in Oregon and Washington, while the other examined patients who sought care for LBP from different HCPs in North Carolina. Two of the RCTs enrolled patients seeking care at health maintenance organizations (HMOs) in California and Washington, while one enrolled patients at a chiropractic college outpatient clinic in Minnesota. All seven studies were focused on LBP, including LBP of any duration (*n* = 4), LBP present for less than 10 weeks (*n* = 1) or 12 weeks (*n* = 1), and LBP for more than 7 days (*n* = 1). Four studies stated they enrolled participants with nonspecific LBP (e.g. LBP of mechanical origin), while four studies excluded participants with potential red flags for serious spinal pathology (e.g. cancer, instability).

**Table 5:** Study design for cost comparisons also examining clinical outcomes

First author	Year	Location/Study design/Eligibility/Study period	Inclusion	Exclusion	Chiropractic group(s)	Clinical outcomes/Maximum follow-up
Bronfort [46]	2000	Minnesota/Pilot randomized controlled trial/Seeking care at chiropractic college clinic/3/1/1998 - 3/1/1999	1. Sciatica; 2. 2–12 weeks duration; 3. Age 20–65; 4. QTF categories 2; 3; 4; 6	1. QTF category 5, 7, or 11; 2. Progressive neurologic deficits; 3. Lumbar surgery; 4. Atherosclerosis or aneurysm; 5. Joint instability; 6. Ankylosing spondylitis; 7. Osteopenia; 8. Blood clotting disorders; 9. Substance abuse; 10. Litigation; 11. Ongoing treatment for LBP; 12. History of gastrointestinal events; 13. Renal insufficiency; 14. Corticosteroids; 15. Average pain score of <30 %; 16. Pregnant or nursing	Chiropractic care (i.e. SMT, massage, traction, self-care instruction from a PT)	Pain, Physical function, Satisfaction/3 months
Carey [40]	1995	North Carolina/Prospective observational/Seeking care from randomly selected HCPs/6/1/1992 - 3/1/1993	1. Low back pain; 2. <10 weeks duration; 3. Spoke English; 4. Own a telephone	1. Previous care for LBP; 2. History of back surgery; 3. History of cancer; 4. Pregnancy	1. Urban chiropractic care	Physical function,
					2. Rural chiropractic care (Both groups received SMT, heat, cold, diathermy, ultrasound, EMS, traction, or OTC drugs)	Satisfaction/6 months
Cherkin [44]	1998	Washington/Comparative randomized controlled trial/Seeking care at 2 primary care clinics with staff-model HMO/11/1/1993 - 9/1/1995	1. Low back pain; 2. >7 days after seeing PCP; 3. Age 20–64; 4. Saw PCP for low back pain	1. Mild or no pain 7 days after initial visit; 2. Back surgery; 3. Sciatica; 4. Systemic or visceral causes of pain; 5. Corticosteroids; 6. Pregnancy; 7. Claims or litigation; 8. Visits to practitioners other than PCPs	Chiropractic care (i.e. SMT, ice, massage, exercise, or manipulation of hip, pelvis, or ischium)	Pain, Physical function,
						Satisfaction/24 months
Haas [41]	2005	Oregon and Washington/Prospective, non-randomized observational/Seeking care from 51 chiropractic clinics and 14 medical clinics/12/8/1994 - 6/30/1996	1. Low back pain of mechanical origin; 2. Acute or chronic; 3. Minimum 18 years of age; 4. English literate; 5. Ambulatory	1. Care from same provider type in previous 6 weeks; 2. Pregnancy; 3. Contraindications to spinal manipulation	Chiropractic care (i.e. SMT, physical modalities, exercise, self-care)	Pain, Physical function,
						Satisfaction/12 months
Kominski [45]	2005	California/Randomized controlled trial/Seeking care at 3 HMOs/10/30/1995 - 11/9/1998	1. Low back pain (+/− leg symptoms); 2. Any duration; 3. Age 18 or older; 4. No treatment for LBP in previous month; 5. 18 month follow-up data available	1. Fracture, tumor, infection, spondyloarthropathy; 2. Cauda equina syndrome or progressive muscle weakness; 3. Severe coexisting condition; 4. Blood coagulation disorder; 5. Planning to relocate; 6. Not easily accessible by telephone; 7. Could not read English; 8. Third-party liability for LBP	1. Chiropractic care (SMT, back care instruction, exercise)	Pain, Physical function/18 months
					2. Chiropractic care + physical modalities (i.e. heat, cold, ultrasound, EMS)	
Sharma [43]	2009	Oregon and Washington/Prospective, non-randomized, observational/Seeking care from 51 chiropractic clinics and 14 medical clinics/12/8/1994 - 6/30/1996	1. Low back pain of mechanical origin; 2. Acute or chronic; 3. Minimum 18 years of age; 4. Ambulatory; 5. English literate	1. Care from same type of provider in previous 6 weeks; 2. Pregnant; 3. Contraindications to spinal manipulation	Chiropractic care (i.e. SMT)	Pain/12 months
Stano [42]	2002	Oregon and Washington/Prospective, non-randomized observational/Seeking care from 51 chiropractic clinics and 14 medical clinics/12/8/1994 - 6/30/1996	1. Low back pain of mechanical origin; 2. Acute or chronic; 3. Minimum 18 years of age; 4. English literate; 5. Complete cost data available; 6. Ambulatory; 7. 1 year VAS and ODI available^a^	1. Pregnancy; 2. Malignancy, infection, vertebral fracture, lumbar instability; 3. Low back surgery in previous year^a^	Chiropractic care (no details reported)	Pain, Physical function/12 months

^a^eligibility criteria reported in separate study [48]

*EMS* electrical muscle stimulation, *ESI* epidural steroid injection, *HCP* health care provider, *HMO* health maintenance organization, *LBP* low back pain, *NSAID* non-steroidal anti-inflammatory drug, *ODI* Oswestry Disability Index, *OTC* over-the-counter, *PCP* primary care provider, *PT* physical therapist, *QTF* Quebec Task Force, *SMT* spinal manipulation therapy, *VAS* visual analog scale

Five studies reported that patients in the chiropractic care groups received a variety of therapies, including SMT, exercise, and physical modalities (e.g. heat, cold, massage, ultrasound, electrical stimulation), while one study stated only SMT and one study did not specify the therapies received. Two of the studies included multiple groups who received chiropractic care (e.g. urban vs. rural chiropractic care, chiropractic care with or without physical modalities). One study had four comparator groups (care from an urban or rural medical physician, care from an orthopedist, or care from a nurse practitioner (NP) or physician’s assistant (PA)), four studies had two comparator groups (care from a medical physician with or without referral to PT or surgeon, care from a PT or educational booklet about LBP, care from a medical physician with or without physical modalities, care from a medical physician or epidural steroid injection), and one study had only one comparator (care from a medical physician).

#### Cost comparison

Cost comparison findings for the seven studies in this group are summarized in Table 6. Four studies estimated health care costs from amounts billed by HCPs, two studies did so from internal cost accounting systems, and one study did not report how health care costs were estimated. All seven studies specified that office visits were included in health care costs; studies also included the costs of diagnostic imaging (*n* = 5), medication (*n* = 3), PT (*n* = 3), surgical care or referral (*n* = 3), and injections (*n* = 2). The number of participants included in groups receiving chiropractic care ranged from 7 to 1,855 (mean 857, SD 768, median 606), while in comparator groups it ranged from 13 to 1,027 (mean 568, SD 387, median 739). The costs of health care for spine pain by participants who received chiropractic care ranged from $214 to $684 (mean $411, SD $194, median $429), while in comparator groups it ranged from $123 to $1,285 (mean $474, SD $401, median $343). In two (29 %) studies, health care costs were lower for patients whose LBP was managed by chiropractic care. The difference in health care costs for members who received chiropractic care ranged from −57 % to 74 % (mean 10 %, SD 40 %, median 8 %).

**Table 6:** Comparison of health care costs in studies also examining clinical outcomes

			Chiropractic care		Comparator group(s)	Comparator		Lowest	Difference^a^
First author	Year	Health care costs included	*n*	Costs		*n*	Costs		
Bronfort [46]	2000	Office visits, injections	7	$550	1. Medical physician care (i.e. prescriptions NSAIDs, acetaminophen, mild narcotic, activity modification, self-care instruction from a PT)	13	$1,285	Chiropractic	−57 %
					2. ESI with activity modification and self-care instruction from a PT				
Carey [40]	1995	Outpatient costs (office visits, radiography, other imaging, medication, PT, other treatment)	606	$684	1. Urban medical physician primary care^b^	1,027	$536	Comparator	28 %
					2. Rural medical physician primary care^b^				
					3. Orthopedist^b^				
					4. HMO provider (i.e. NPs and PAs)				
Cherkin [44]	1998	Office visits, imaging, laboratory tests, medications	122	$429	1. PT care (i.e. exercise, lumbar cushion support, McKenzie book, education)	199	$343	Comparator	25 %
					2. Educational booklet (i.e. The Back Book)				
Haas [41]	2005	Office visits, advanced imaging, surgical consultation, PT	1,855	$222	Medical physician care (i.e. prescription drugs, exercise, self-care advice, PT referral)	925	$211	Comparator	5 %
Kominski [45]	2005	Outpatient costs (office visits, surgery, injection, other)	325	$558	1. Medical physician care (i.e. back care advice, exercise, bed rest, narcotic analgesics, muscle relaxants, anti-inflammatories, OTC pain relievers)	329	$616	Chiropractic	−10 %
					2. Medical physician care + physical modalities (i.e. heat, cold, ultrasound, EMS, soft tissue and joint mobilization, mechanical traction, SET)				
Sharma [43]	2009	Office visits, advanced imaging, surgical consultation, PT	1,558	$220	Medical physician care	744	$205	Comparator	8 %
Stano [42]	2002	Office visits, medication, radiography	1,524	$214	1. Medical physician care + referral to surgeon and/or PT	739	$123	Comparator	74 %
					2. Medical physician care without referral to surgeon or PT				

^a^reported as (−(comparator costs - chiropractic care costs)/comparator costs) × 100 %

^b^no details reported about care received

*EMS* electrical muscle stimulation, *ESI* epidural steroid injection, *HMO* health maintenance organization, *NP* nurse practitioner, *OTC* over-the-counter, *PA* physician assistant, *PT* physical therapist, *SET* supervised exercise therapy

#### Study quality assessment

Assessment of methodological quality for the seven studies in this group is summarized in Table 7. The number of questions on the CHEC list that could be answered “yes” ranged from 7 to 14 (mean 10.1, SD 2.3, median 10). The questions most commonly scored as “yes” were items 4, 11, 16, and 18, which were present in all seven studies. The questions most commonly scored as “no” items were items 12, 14, and 19, none of which were present in any of the seven studies.

**Table 7:** Quality assessment of cost comparison studies also examining clinical outcomes

#	Question	Bronfort, 2000 [46]	Carey, 1995 [40]	Cherkin, 1998 [44]	Haas, 2005 [41]	Kominski, 2005 [45]	Sharma, 2009 [43]	Stano, 2002 [42]	Total
1	Is the study population clearly described?	yes	no	yes	yes	no	yes	yes	5
2	Are competing alternatives clearly described?	yes	no	yes	no	yes	no	no	3
3	Is a well-defined research question posed in answerable form?	yes	yes	yes	yes	yes	yes	no	6
4	Is the economic study design appropriate to the stated objective?	yes	yes	yes	yes	yes	yes	yes	7
5	Is the chosen time horizon appropriate in order to include relevant costs and consequences?	no	no	yes	yes	yes	yes	yes	5
6	Is the actual perspective chosen appropriate?	no	no	yes	no	yes	no	no	2
7	Are all important and relevant costs for each alternative identified?	no	yes	yes	no	yes	no	no	3
8	Are all costs measured appropriately in physical units?	no	yes	yes	no	yes	no	no	3
9	Are costs valued appropriately?	no	no	yes	no	no	no	no	1
10	Are all important and relevant outcomes for each alternative identified?	yes	no	yes	yes	yes	yes	yes	6
11	Are all outcomes measured appropriately?	yes	yes	yes	yes	yes	yes	yes	7
12	Are outcomes valued appropriately?	no	no	no	no	no	no	no	0
13	Is an incremental analysis of costs and outcomes of alternatives performed?	no	no	no	yes	no	no	yes	2
14	Are all future costs and outcomes discounted appropriately?^a^	N/A	N/A	N/A	N/A	N/A	N/A	N/A	N/A
15	Are all important variables, whose values are uncertain, appropriately subjected to sensitivity analysis?	no	no	no	no	no	yes	no	1
16	Do the conclusions follow from the data reported?	yes	yes	yes	yes	yes	yes	yes	7
17	Does the study discuss the generalizability of the results to other settings and patient/client groups?	yes	no	yes	yes	yes	yes	yes	6
18	Does the article indicate that there is no potential conflict of interest of study researcher(s) and funder(s)?	yes	yes	yes	yes	yes	yes	yes	7
19	Are ethical and distributional issues discussed appropriately?	no	no	no	no	no	no	no	0
	Total	9	7	14	10	12	10	9	

^a^not applicable (i.e. studies did not project future costs or health outcomes)

*N/A* not applicable

#### Comparison of clinical outcomes

The clinical outcomes examined in these seven studies included pain (*n* = 6), physical function (*n* = 6), and patient satisfaction (*n* = 4). Among the six studies that measured pain, only two reported significant differences between the groups compared. In one study, participants who received chiropractic care had greater improvement in pain than those who received care from a medical physician after both 3 months and 12 months [41]. In another study, chiropractic care had greater improvement in pain than an educational booklet about LBP after 1 month, but not after 3, 12, or 24 months; after adjusting for baseline variables, the difference in pain after 1 month was no longer significant [44]. One study did not report differences in pain between groups due to the limited sample size (i.e. *n* = 20) [46].

In the six studies that measured physical function, only one study reported significant differences between the groups compared. In that study, participants who received chiropractic care had greater improvement in physical function after both 3 months and 12 months than those who received care from a medical physician [41]. Three of the four studies that measured patient satisfaction reported differences between the groups compared. In two studies, participants who received chiropractic care were more satisfied than those who received care from a medical physician [40, 41]. In another study, participants who received chiropractic care (or care from a PT) were more satisfied than those who received an educational booklet about LBP [44]. One study did not report differences in satisfaction between groups due to the limited sample size (i.e. *n* = 20) [46].

### Adjusted vs. unadjusted cost comparisons

Ten studies (42 %) reported adjusting their comparison of health care costs to account for differences that may have existed between study groups in sociodemographics or clinical factors (e.g. patients receiving chiropractic care having less severe symptoms than those receiving care from a medical physician); their findings are summarized in Table 8 [22–24, 28, 31, 32, 39–41, 45]. The most commonly used factors to adjust these findings included patient location (*n* = 5), age (*n* = 4), gender (*n* = 4), physical function (*n* = 3), pain (*n* = 3), type of health insurance (*n* = 3), and comorbidities (*n* = 3). Before adjusting for various factors, 6/10 (60 %) studies reported that health care costs for spine pain were lower with chiropractic care than comparator groups; this proportion remained constant after adjusting for differences between groups. However, one study that reported unadjusted health care costs were lower with chiropractic care noted that adjusted health care costs were in fact lower for the comparator groups, and another study reported the opposite.

**Table 8:** Adjusted vs. unadjusted cost comparisons

			Unadjusted health care costs	Comparator groups(s)			Adjusted health care costs			
First author	Year	Risk adjustment variables	Chiropractic		Comparator	Lowest	Chiropractic	Comparator	Lowest	Comments
Butler [39]	2010	Physical function, back pain severity, leg pain severity	$868	1. MD care, 2 MD care + chiropractic care +/− PT care, 3. MD care + PT care, 4. Other	$264	Comparator	$6,984	$8,108	Chiropractic	
Carey [40]	1995	Physical function, sciatica, income, duration of pain, worker’s compensation	$684	1. Urban medical physician primary care^a^	$536	Comparator	$699	$523	Comparator	
				2. Rural medical physician primary care^a^						
				3. Orthopedist^a^						
				4. HMO provider (i.e. NPs and PAs)						
Haas [41]	2005	Health insurance, marital status, income	$222	Medical physician care (i.e. prescription drugs, exercise, self-care advice, PT referral)	$211	Comparator	N/A	N/A	Comparator	Adjusted costs were $14 higher for chiropractic care
Kominski [45]	2005	Demographics, physical function, pain, copayments	$558	1. Medical physician care (i.e. back care advice, exercise, bed rest, narcotic analgesics, muscle relaxants, anti-inflammatories, OTC pain relievers)	$616	Chiropractic	$570	$567	Comparator	
				2. Medical physician care + physical modalities (i.e. heat, cold, ultrasound, EMS, soft tissue and joint mobilization, mechanical traction, SET)						
Liliedahl [32]	2010	Symmetry Pharmacy Risk Groups (pharmacy claims, age, and sex)	$756	MD care	$1,037	Chiropractic	$533	$661	Chiropractic	
Shekelle [31]	1995	Sociodemographics, health status, attitude, insurance status, location	$264	1. MD primary care, 2. orthopedist, 3. internist, 4. DO, 5. other providers	$166	Comparator	N/A	N/A	Comparator	Adjusting changed estimates by $13 but did not change rank order of costs
Stano [28]	1996	SysteMetrics classification	$518	MD care	$1,020	Chiropractic	N/A	N/A	Chiropractic	Risk adjusting did not change differences between groups
Stano [23]	1995	SysteMetrics classification, age, sex, location, employee/dependent, insurance type, coinsurance, deductible, type of chiropractic coverage	$493	MD care	$1,000	Chiropractic	$508	$542	Chiropractic	
Stano [24]	1994	Age, sex, region, work status, employee/dependent, health plan type	$5,474	MD care	$8,427	Chiropractic	N/A	N/A	Chiropractic	Adjusted costs were $1,197 lower for chiropractic care
Stano [22]	1993	Age, sex, location, employee/dependent, work status, coinsurance, deductible, health plan coverage of chiropractic	$2,150	MD care	$3,127	Chiropractic	N/A	N/A	Chiropractic	Adjusted costs were 27 % lower for chiropractic care

^a^no details reported about care received

*DO* doctor of osteopathy, *EMS* electrical muscle stimulation, *HMO* health maintenance organization, *MD* medical doctor, *N/A* not applicable, *NP* nurse practitioner, *OTC* over-the-counter, *PA* physician assistant, *PT* physical therapist, *SET* supervised exercise therapy

## Discussion

This review identified 25 cost comparison studies published in English since 1993 that were related to chiropractic care for spine pain in the US. The largest group of studies examined data from private health plans (*n* = 12), while smaller groups of studies examined data from WC plans (*n* = 6), or also examined clinical outcomes (*n* = 7). There were notable differences in study design not only between these three groups of studies, but also among studies within these groups; each group is briefly discussed below.

Overall, 11/12 (92 %) studies in private health plans reported that health care costs were lower for members whose spine pain was managed by chiropractic care, by a mean of 36 %. It should be noted that the only study reporting higher health care costs with chiropractic care was also the only study to examine costs billed by HCPs rather than costs allowed or paid by health plans. It is unknown if differences that may exist in the amounts billed, allowed, and paid by HCPs may have influenced this finding (e.g. DCs may bill more but be paid a smaller amount) [31]. It should also be noted that 7/12 (58 %) studies in this group were conducted by the same author (Miron Stano, PhD) and appeared to use the same source for private health plan claims data (MEDSTAT), raising the possibility of duplicate or unusually homogeneous study findings.

Differences in studies examining data from private health plans were found in the type of spine pain (e.g. LBP only, all regions of spine pain), definition of spine pain (e.g. number of ICD-9 codes included), size of the population size studied, length of claims data (e.g. 12 vs. 24 vs. 48 months), age of claims data (e.g. from 1988 to 2006), focus on members or episodes of care, definition of episodes of care (e.g. no care for 35 vs. 90 days), assignment of health care costs to HCPs (e.g. first HCP seen vs. HCP who gave majority of care), focus on patients with single vs. multiple episodes of care, scope of health care costs (e.g. all conditions vs. spine only), and categories of health care costs examined (e.g. office visits/outpatient care/inpatient care/medications). Not surprisingly, large differences were therefore found in health care costs for both members who received chiropractic care (lowest $264, highest $6,171) or comparator groups (lowest $166, highest $9,958). Furthermore, few details were provided about the actual care received for spine pain from different HCPs. For example, while some readers may assume that chiropractic care consisted primarily of guideline-endorsed therapies such as SMT, patient education, and supervised exercise, such care could also have been provided by medical physicians, PTs, or other HCPs, making comparisons difficult based on the type of HCP seen.

Similar differences were also noted in the design of six cost comparison studies examining data from WC plans, as well as the inclusion or exclusion of patients who underwent spine surgery, examination of open vs closed WC claims, adherence to the different HCPs compared (e.g. patients who remained loyal to one type of HCP vs. those who switched), types of WC claims (e.g. medical only vs. temporary or permanent disability), and examination of health care costs both during and after the period of disability (e.g. so-called “maintenance” care). Although health care costs in groups who received chiropractic care were somewhat similar across these six studies (lowest $415, highest $1,296), large differences were noted in the comparator groups (lowest $264, highest $7,904). Few details were provided about how state regulations of health care for WC claims may have impacted differences in health care costs (e.g. period during which employer has choice of HCPs vs. employee, proportion of earnings covered while on disability). It is also important to note that while indirect costs (e.g. lost productivity) account for a majority of the total costs of spine pain they are difficult to measure and often complex. Only 5/25 (20 %) measured these costs; they were generally lower for patients receiving chiropractic care [33, 35, 36, 38].

It is interesting that most cost comparison studies based on administrative claims data (e.g. private health plans or WC plans) reported that health care costs were generally lower for members/workers whose spine pain was managed with chiropractic care. However, in studies that also examined clinical outcomes and therefore had richer sources of clinical data than studies based only on administrative claims, the reverse was noted and health care costs were generally higher among patients who received chiropractic care. It is unknown if this difference is related to the intensity or type of health care received in pragmatic “real-world” studies (e.g. retrospective examination of administrative claims data) when compared to prospective, comparative cohorts or RCTs in which HCPs attempt to follow specific treatment protocols. Somewhat less variation was noted among these seven studies in the health care costs of participants who received chiropractic care (lowest $214, highest $684) or comparators (lowest $123, highest $1,285) than studies examining private health plans or WC plans.

Few health economic evaluations (HEEs) reported the incremental cost-effectiveness ratios (ICERs) for pain, function, and patient satisfaction, despite renewed interest in such measures to help determine the value of various health care interventions. Although the ICERs that were estimated from the data reported appeared quite small (i.e. $1-10 per 1-point difference in 0–10 visual analog scale (VAS)), the willingness to pay (WTP) for such outcomes in patients with spine pain is unclear. It may be worthwhile to explore various WTP thresholds for such measures from different perspectives (including the patient’s) in future economic evaluations, perhaps as part of efforts to implement value-based health benefits insurance design for spine pain [51].

The methodological quality of the seven studies also examining clinical outcomes was suboptimal, with 8/19 (42 %) items on the CHEC instrument being absent in a majority of studies. No apparent relationship was observed between methodological quality and differences in health care costs reported between study groups. It should be noted that only one study scored a “yes” on item 9 “Are costs valued appropriately?” and all studies scored a “no” on item 12, “Are outcomes valued appropriately?” This is a limitation that should be addressed in future evaluations as only one study based costs on resource utilization and no study provided health-related utility estimates.

Generally few differences were reported between study groups in the efficacy of different approaches to managing spine pain, whether chiropractic care, care from a medical physician, care from a PT, or an educational booklet about LBP. These findings are consistent with conclusions from recent systematic reviews suggesting that the efficacy of SMT (e.g. most commonly used by chiropractors) for acute and chronic LBP is likely comparable to that of other recommended conservative approaches, including non-steroidal anti-inflammatory drugs (NSAIDs), analgesics, or self-care [52, 53]. Previous reviews have also highlighted the methodological weaknesses of economic evaluations related to spine pain, concluding that the health outcomes achieved with chiropractic care were similar to various comparators, with small differences in costs [12, 14, 54].

In general, the findings in this review suggest that health care costs may be somewhat lower when spine pain is managed with chiropractic care in the US, even if such differences are sometimes attributable to sociodemographics, clinical, or other factors rather than HCPs. These findings echo that of a review published in 1993 that examined studies in which LBP was managed by SMT, chiropractic care, other interventions (e.g. physical modalities, medications, exercise) throughout the world (e.g. Australia, Canada, Egypt, Italy, the Netherlands, New Zealand, Nigeria, Sweden, United Kingdom, and US) [11]. Based on the favorable short-term clinical improvements and lower costs of care reported in those studies, the previous review concluded that health care costs could be reduced if a higher proportion of patients with spine pain received chiropractic care rather than other interventions, and recommended a greater integration of chiropractors into the publicly financed health care system in Ontario, Canada. However, that recommendation was never implemented, and publicly financed coverage of chiropractic services (and other health care services) was subsequently eliminated in Ontario to alleviate budget deficits [55]. A more recent review was published on the clinical and economic evidence on chiropractic care for the management of LBP in 2005 [56]. The study found that although outcomes were similar between chiropractic care and standard medical care, the evidence remained inconclusive for costs.

Recent studies have assessed the efficacy of integrating chiropractic care for spine pain into the mainstream health care system with mixed results. For example, a study in Vancouver, Canada, compared the clinical outcomes of patients who received evidence-based care, including chiropractic care, for LBP in a hospital setting to usual care from a primary care provider (PCP) [57]. Findings suggested that usual care from a PCP was rarely evidence-based, with many patients receiving bed rest, opioids, and passive physical modalities, and few receiving exercise and reassurance. After 6 months, patients who received chiropractic care were more likely to improve than those receiving usual care from a PCP; costs were not measured. Other studies have reported similarly favorable clinical or economic results in both Canada (i.e. Calgary, Ottawa) and the US (i.e. Boston) [58–61]. However, a recent study that increased Medicare coverage of chiropractic care to allow additional health services (e.g. physical modalities and procedures, x-rays, referrals for magnetic resonance imaging (MRI)) and diagnoses (e.g. neuromuscular conditions) in parts of Maine, New Mexico, northern Illinois, Iowa, and Virginia reported that this increased total health care costs [62]. Although a subsequent analysis reported that the vast majority of cost-increases occurred in only one of the demonstration areas (i.e. northern Illinois), the demonstration project was not deemed successful and the Centers for Medicare and Medicaid Services (CMS) did not pursue expanded coverage of chiropractic care to reduce health care costs [63].

### Limitations

There are several limitations to this review, which only examined studies published in English conducted in the US, limiting its generalizability in other settings. As noted above, the studies reviewed differed widely in their methodology, which presents challenges when interpreting and comparing their findings. Studies evaluated in the manuscript only evaluated costs from a third party payer perspective. Whereas evaluations from a societal and governmental perspective are generally preferable, few studies have access to such comprehensive data when comparing costs.

Although health care costs from the studies included could have been presented in constant 2015 dollars using published US health care inflation factors, doing so over an extended period (e.g. 1988 to 2006) would likely have masked other important differences in US health care costs during that time, including changes in health plan types (e.g. fee for service vs. HMO), therapies used to manage spine pain (e.g. passive vs. active care), health plan coverage of therapies (e.g. motorized traction therapy), fees for specific therapies (e.g. bundled vs. itemized billing), health plan cost sharing (e.g. copays, coinsurance, deductibles), coverage limits (e.g. annual visits to specific HCPs), and cost containment methods (e.g. prior authorization). Therefore, the original costs reported in each study are presented in this review and as such, are not easily compared across studies. In addition, this review aggregated data to present single estimates related to health care costs for those who received chiropractic care or other comparators, which may have masked important differences noted in study subgroups. Three of the authors are trained as chiropractors, which may create some bias, and were formerly consultants of a specialty managed care company in the US (Palladian Health).

## Conclusions

This review identified 25 cost comparison studies related to chiropractic care for spine pain in the US and published in English since 1993. Although findings from the studies reviewed generally suggested that chiropractic care may be associated with lower health care costs when compared to care from other HCPs, the methods used in these studies differed widely, limiting their interpretation and generalizability. Additional research using more rigorous methods is needed to determine if differences in health care costs noted in these studies are attributable to the type of health care received for spine pain or patient sociodemographic, clinical, or other factors that may be unrelated to health care.

## Additional file

Additional file 1:
**Medline search strategy.** (DOCX 14 kb)

## Abbreviations

US: United States
HCP: Health care provider
WC: Workers’ compensation
CHEC: Consensus on health economic criteria
MD: Medical doctor
DO: Doctor of osteopathy
PT: Physical therapist
LBP: Low back pain
SMT: Spinal manipulation therapy
CPG: Clinical practice guideline
CAM: Complementary and alternative medical
EED: Economic evaluation database
HTA: Health technology assessment
ICL: Index to chiropractic literature
DC: Doctor of chiropractic
HMO: Health maintenance organizations
ICD: International classification of diseases
SD: Standard deviation
OBS: Observational
RCT: Randomized controlled trials
NP: Nurse practitioner
PA: Physician’s assistant
HEE: Health economic evaluations
ICER: Incremental cost-effectiveness ratio
VAS: Visual analog scale
WTP: Willingness to pay
NSAID: Non-steroidal anti-inflammatory drug
PCP: Primary care provider
MRI: Magnetic resonance imaging
CMS: Centers for Medicare and Medicaid services
