# Supplementary material for: A systematic review comparing the costs of chiropractic care to other interventions for spine pain in the United States
Source: BMC Health Serv Res. 2015 Oct 19;15:474. doi: 10.1186/s12913-015-1140-5 (PMC4615617; doi:10.1186/s12913-015-1140-5)
Supplement: Additional file 1: — Medline search strategy. (DOCX 14 kb) [file 12913_2015_1140_MOESM1_ESM.docx]

**Appendix 1. Medline search strategy**

*Chiropractic care*

| 1 | exp | chiropractic |  |
| --- | --- | --- | --- |
| 2 | exp | manipulation, chiropractic |  |
| 3 | exp | manipulation, spinal |  |
| 4 |  | chiropractor | .mp |
| 5 |  | chiropractic | .mp |
| 6 |  | chiropra* | .mp |
| 7 |  | spin* manipulati* | .mp |
| 8 |  | spin* mobili*ation | .mp |
| 9 |  | manual therap* | .mp |
| 10 |  | manipulati* therap* | .mp |
| 11 |  | spin* adjust* | .mp |
| 12 |  | or/1-11 |  |

*Cochrane Back Review Group (CBRG) part B - Specific search for thoracic, low back, sacrum and coccyx problems*

| 1 |  | dorsalgia | ti,ab |
| --- | --- | --- | --- |
| 2 | exp | Back Pain/ |  |
| 3 |  | backache | ti,ab |
| 4 | exp | low back pain |  |
| 5 |  | (lumbar adj pain) | ti,ab |
| 6 |  | coccyx | ti,ab |
| 7 |  | coccydynia | ti,ab |
| 8 |  | sciatica | ti,ab |
| 9 | exp | sciatic neuropathy |  |
| 10 |  | spondylosis | ti,ab |
| 11 |  | lumbago | ti,ab |
| 12 |  | back disorder$ | ti,ab |
| 13 |  | or/1-12 |  |

*CBRG part C - Specific search for neck problems*

| 1 | exp | neck muscles |  |
| --- | --- | --- | --- |
| 2 | exp | Neck/ |  |
| 3 | exp | neck pain/ |  |
| 4 | exp | whiplash injuries |  |
| 5 |  | neck | ti,ab |
| 6 |  | or/1-5 |  |

*CBRG part D - Other spinal disorders*

| 1 | exp | Spine/ |  |
| --- | --- | --- | --- |
| 2 |  | discitis | ti,ab |
| 3 | exp | Spinal Diseases/ |  |
| 4 |  | (disc adj degeneration) | ti,ab |
| 5 |  | (disc adj prolapse) | ti,ab |
| 6 |  | (disc adj herniation) | ti,ab |
| 7 | exp | spinal fusion |  |
| 8 | exp | spinal neoplasms |  |
| 9 |  | (facet adj joints) | ti,ab |
| 10 | exp | intervertebral disc |  |
| 11 | exp | intervertebral disc displacement |  |
| 12 |  | postlaminectomy | ti,ab |
| 13 |  | arachnoiditis | ti,ab |
| 14 |  | (failed adj back) | ti,ab |
| 15 |  | or/1-14 |  |

*CBRG part E - Specific outcome measurements related to spinal disorders*

| 1 |  | oswestry | .tw |
| --- | --- | --- | --- |
| 2 |  | roland-morris | .tw |
| 3 |  | or/1-2 |  |

*Economic evaluations*

| 1 | exp | "Costs and cost analysis" |  |
| --- | --- | --- | --- |
| 2 | exp | Economics, Medical/ |  |
| 3 | exp | Insurance, health |  |
| 4 | exp | "Fees and charges" |  |
| 5 | exp | Managed Care Programs |  |
| 6 | exp | Quality-Adjusted Life Years/ |  |
| 7 |  | cost effectiveness | .mp |
| 8 |  | cost utility | .mp |
| 9 |  | cost benefit | .mp |
| 10 |  | cost minimization | .mp |
| 11 |  | cost consequence | .mp |
| 12 |  | cost comparison | .mp |
| 13 |  | economic evaluation | .mp |
| 14 |  | qaly | .mp |
| 15 |  | cba | .mp |
| 16 |  | cea | .mp |
| 17 |  | cua | .mp |
| 18 |  | budget* | .mp |
| 19 |  | expenditure* | .mp |
| 20 |  | price* | .mp |
| 21 |  | or/1-21 |  |
